# Nutritional risk markers among stroke out-patients at the neurology clinic of a teaching hospital in Ghana

**DOI:** 10.11604/pamj.2020.37.258.16929

**Published:** 2020-11-23

**Authors:** Lloyd Chauwa, Collins Afriyie Appiah, Kwabena Nsiah, Fred Stephen Sarfo

**Affiliations:** 1 Department of Biochemistry and Biotechnology, Faculty of Biosciences, College of Science, Kwame Nkrumah University of Science and Technology, Kumasi, Ghana,; 2 Department of Human Nutrition and Health, Faculty of Food and Human Sciences, Lilongwe University of Agriculture and Natural Resources, Lilongwe, Malawi,; 3 School of Medical Sciences, Kwame Nkrumah University of Science and Technology, Kumasi, Ghana,; 4 Neurology Unit, Department of Medicine, Komfo Anokye Teaching Hospital, Kumasi, Ghana

**Keywords:** Stroke survivors, malnutrition, anaemia, body mass index, MUAC

## Abstract

**Introduction:**

stroke survivors are at risk of malnutrition due to inadequate dietary intake, as a result of neurological disorders causing dysphagia, depression and impaired ability to self-feed. There is paucity of information on nutritional status of stroke survivors after discharge from hospital care, hence, this study sought to determine the nutritional risk markers among stroke out-patients at the Neurology Clinic of Komfo Anokye Teaching Hospital, Kumasi, Ghana.

**Methods:**

a cross-sectional study was conducted among 106 stroke survivors at Komfo Anokye Teaching Hospital, Kumasi, Ghana. Nutritional status of stroke survivors was assessed, using body mass index (BMI) and mid upper arm circumference (MUAC). Biochemical and haematological nutrition indicators including total serum protein, serum albumin, total lymphocyte count, uric acid and haemoglobin were also determined. Independent t-test and ANOVA were used to test differences between mean values.

**Results:**

the mean age of study participants was 58.47±14.2 years, with 56% being females. Overall, 96 (88.7%) of the participants had malnutrition, of whom 66 (68.8%) were undernourished, while 30 (31.2%) had overnutrition. It was also found that 38.7% of the participants were anaemic, based on haemoglobin levels. Using mean BMI, stroke survivors who had been discharged over five years were significantly overweight (p = 0.010).

**Conclusion:**

there was high level of malnutrition among stroke out-patients in this study. The most common nutrition-related problem in the stroke survivors studied was anaemia. Findings from this study suggest the need for nutrition intervention strategies to address the high burden of malnutrition among the stroke survivors.

## Introduction

According to the World Health Organization (WHO), stroke is defined as a clinical condition presumed to be of vascular origin, characterized by rapid development of focal or global disturbance of cerebral functions lasting for 24 hours or longer, leading to morbidity or death [1]. Stroke is the second leading cause of disability in adult life, as over 50% of the survivors are rendered disabled, while 25% of them tend to develop dementia [2]. The burden of stroke, a non-communicable disease (NCD), is rapidly increasing in low and middle-income countries including those in sub-Saharan Africa [3-5]. Stroke survivors are at a heightened risk of protein-energy malnutrition due to inadequate dietary intake, as a result of neurological disorders causing dysphagia, depression and impaired ability to self-feed [6]. Though nutrition is fundamental in preventing as well as recovering from stroke, it is under-appreciated by many physicians and other health caregivers [7]. For enhanced recovery, stroke survivors require adequate energy intake, adequate protein, right amount of fat and all other essential nutrients, given that there is high nutrient demand in the rehabilitation phase, due to oxidative stress [8]. It is therefore, important to identify and treat the decline in nutritional status associated with stroke since it tends to have an impact on the functional recovery and survival [6].

Among stroke survivors, it has been shown that the three-month period after discharge from hospital is the riskiest period for the survivors and about 30% tend to die within this time due to inadequate care [9]. Any acute stroke survivor, who has been discharged from hospital is at an increased risk of developing undernutrition, which may lead to poor functional capacity if proper nutrition, physical exercise, controlling comorbid conditions such as hypertension and compliance to medical treatment are not done [10].

In the Ashanti Region, Ghana, stroke contributes 9.1% to total hospital admissions and also contributes 13.2% per year to all medical adult deaths in people aged sixty (60) years and above [11]. Despite the increasing prevalence, there is no information on nutritional status of outpatient stroke survivors, hence the need for this study. The level of serum albumin, serum transferrin, haemoglobin and total lymphocyte count can be used to evaluate nutritional status, which has direct impact on recovery and re-gaining of functionality [12]. Given the paucity of data on nutritional status of stroke survivors in sub-Saharan Africa, this study sought to determine the nutritional risk markers of stroke survivors, attending an out-patient neurology clinic at Komfo Anokye Teaching Hospital, Kumasi, Ghana.

## Methods

**Participants:** in this cross-sectional study, one hundred and six (106) participants, aged 18 years and above were enrolled from out-patient stroke survivors who were attending scheduled medical reviews at the Komfo Anokye Teaching Hospital in Kumasi Metropolis, Ghana.

### Data collection

**Socio-demographic data:** socio-demographic data and medical history of the participants were assessed, using a structured questionnaire.

**Anthropometric data:** anthropometric measurements (mid upper arm circumference, height and weight) of the participants were determined, using inelastic measuring tape, weighing scale (Soehnle, Germany) and stadiometer (Secca 213, Germany). The weight measurement was done, while the participants were in light clothes with no shoes, while standing straight with arms placed along the body and the weight was recorded to nearest 0.1kg. Standing height was measured in meters, using a stadiometer (Secca 213, Germany) in meters. Body mass index was calculated from the weight and height measurements (weight/height in m^2^).

Mid upper arm circumference (MUAC) was measured in centimeters, using inelastic measuring tape. The measurement was taken on the left arm between the acromion process of the humerus and olecranon process of ulna, while the arm was relaxed alongside the body. The MUAC was used to estimate muscle mass, as an indicator of protein stores [6].

**Collection of blood samples from participants for analyses of nutrition indicators:** five milliliters of venous blood sample were collected from each participant for determination of biochemical/ haematological nutrition indicators (total serum protein, serum albumin, total lymphocyte count, uric acid and haemoglobin). Uric acid has been included in this study, based on the premise that dietary factors may influence serum levels by providing purines as precursors of uric acid, increasing or decreasing nucleotide turnover or by influencing its excretion [13].

**Determination of nutritional status of stroke survivors:** the determination of nutritional status of the stroke survivors was based on two anthropometric, three biochemical and two haematological indicators [13]. If two or more of these indicators were out of the normal range, the study participant was classified as malnourished, while any participant who had all the nutritional indicators within normal range, or just one indicator out of the normal range, was classified as well-nourished [12,13]. The nutritional status was further categorized into three, namely: under-nutrition, well-nourished and over-nutrition. A person was regarded to have under-nutrition if two or more of the nutritional indicators were on the lower side of the normal range, while those who had their nutritional indicators above the normal range were regarded to have overnutrition [12].

**Ethical approval:** ethical approval was obtained from the Committee on Human Research, Publications and Ethics of Kwame Nkrumah University of Science and Technology (KNUST), Kumasi (Reference number: CHRPE/AP/406/16). The stroke survivors and caregivers were made aware of the aim of the study, then written informed consent was obtained from them, prior to the interview and data collection.

**Statistical analysis:** Statistical Package for Social Sciences (SPSS) software, version 23 was used for analysis. Microsoft Excel software was used to generate tables and figures (graphs). The means and standard deviations were calculated for continuous variables, while frequencies and percentages were calculated for categorical variables. Independent t-test was used to test differences between two mean values; while Analysis of Variance (ANOVA) was used to test differences among mean values of more than two groups.

## Results

**Demographic characteristics of the stroke survivors:** the study population comprised 106 stroke survivors. Their mean age was 58.5±14.2 years; the minimum age was 21, while the maximum was 94 years. There was a slight preponderance of females who made up 56% of the study sample. Survivors who were more than 60 years predominated (47%) followed by those who were 51 to 60 years (26%). The participants who were 51 years and above constituted 73%. The majority, 82%, of the participants were married. With regard to educational status, majority of the participants (56%) had middle school education, while 4% had tertiary education (Table 1).

**Table 1:** demographic information on stroke survivors

Categories	N(%)
**Sex**	
Male	47(44)
Female	59(56)
Total	106(100)
**Age (years)**	
20-29	3(3)
30-39	8(8)
40-49	17(16)
50-59	28(26)
60+	50(47)
Total	106(100)
**Marital status**	
Single	5(5)
Married	87(82)
Divorced	9(8)
Widow	5(5)
Total	106(100)
**Educational level^a^**	
Low	42(40)
Middle	60(56)
High	4(4)
Total	106(100)
**Religion**	
Christian	98(92)
Muslim	8(8)
Total	106(100)
**Tribe**	
Asante	92(87)
Others	14(13)
Total	106(100)

Educational level^a^: low = no school and primary; middle = JHS and SHS; high = tertiary education

**Type and number of stroke episodes among stroke survivors:** from Table 2, participants who had experienced ischaemic stroke (66%) were more than those who had haemorrhagic stroke (34%). Most of the stroke survivors (91%) had their first-time episode of stroke. The period of discharge from the hospital ranged from 6 months to 17 years and the mean period after discharge from in-patient hospital care was 3.31 years.

**Table 2:** age of patients, years after discharge, type and number of stroke episodes

Variable	N (%)	
**Type of stroke**		
Ischaemic	70 (66)	
Haemorrhagic	36 (34)	
Total	106 (100)	
**Number of stroke episodes**		
Once	96 (91)	
More than once	10 (9)	
Total	106 (100)	
	**Mean (**±**SD)**	**95% CI**
Age of Survivors (years)	58.47±14.2	21-94
Period after discharge (years)	3.31±3.5	0.6-17

CI=confidence interval

**Nutritional assessment of stroke survivors:** the nutrition assessment included two anthropometric measurements (MUAC and BMI), three biochemical indicators (uric acid, serum albumin, total protein) and the two haematological indices (total lymphocyte count, haemoglobin). Based on MUAC, 57.5% of the participants were well nourished and 34.9% were underweight, whilst 45.3% were classified as normal weight 26.4% were underweight according to BMI. The prevalence of obesity according to MUAC and BMI was 7.5% and 12.3% respectively (Table 3).

**Table 3:** anthropometric measurements of stroke survivors

Anthropometric measurement	Nutritional status (categories)	Overall n=106(%)	Females n=59(%)	Males n=47(%)
BMI	Underweight	28(26.4)	14(23.7)	14(29.8)
	Normal weight	48(45.3)	26(44.1)	22(46.8)
	Overweight	17(16.0)	9(15.3)	8(17.0)
	Obese	13(12.3)	10(16.9)	3(6.4)
MUAC	Underweight	37(34.9)	22(37.3)	15(31.9)
	Well nourished	61(57.5)	30(50.8)	31(66.0)
	Obese	8(7.5)	7(11.9)	1(2.1)

BMI: underweight=<18.5kg/m^2^, normal weight=18.5-24.9kg/m^2^, overweight=25.0-29.9kg/m^2^ and obesity=≥30.0kg/m^2^ (WHO, 1998); MUAC (cm): underweight=<23.0cm, well-nourished=23.5-33.0cm and obese=>33cm (WHO, 1998)

**Biochemical and haematological nutrition markers:** as summarized in Table 4, majority of the participants had normal albumin levels and only 2.1% had low albumin level. The total protein showed a similar trend, as the participants with normal levels were the majority. Regarding uric acid levels, 25.5% of the participants recorded values that were higher than the reference range. Based on haemoglobin levels, 38.7% of the participants were anaemic. Participants with low lymphocyte count constituted 17% of the study participants.

**Table 4:** levels of biochemical markers among the stroke survivors

Biochemical marker	Male: N=47			Female: N=59			Overall: N=106		
	Low (%)	Normal (%)	High (%)	Low (%)	Normal (%)	High (%)	Low (%)	Normal (%)	High (%)
Serum albumin (g/L)	2.1	97.9	0.0	5.1	94.9	0.0	3.8	96.2	0.0
Uric acid (μmol/l)	6.4	68.1	25.5	10.2	54.2	35.6	8.5	60.4	31.1
Total lymphocytes count (mm^3^)	17.0	83.0	0.0	6.8	93.2	0.0	11.3	88.7	0.0
Haemoglobin (Hb)-(g/dL)	42.6	57.4	0.0	35.6	64.4	0.0	38.7	61.3	0.0
Total protein (g/L)	4.3	89.4	6.4	1.7	89.8	8.5	2.8	89.6	7.5

Serum albumin (g/L): low=<30g/L, normal=30-55g/L and high=>55g/L; uric acid (μmol/L): male {low=<202μmol/l, normal=202-416μmol/l and high=>416umol/l} female: {low=<142umol/l, normal=142-330umol/l and high=>330umol/l}; total lymphocytes count (mm^3^): low=<1500mm^3^ and normal=>1500ul/mm^3^; haemoglobin (Hb)-(g/dL): Male {low=<12.5g/dl, normal=12.18g/dl, high=>18.8g/dl: female: {low=<11.5g/dL, normal=11.5-16.5g/dl, high=>16.5g/dl; total protein (g/l): low=<60g/l, normal=60-80g/l, high=>80g/l

**Figure 1:**
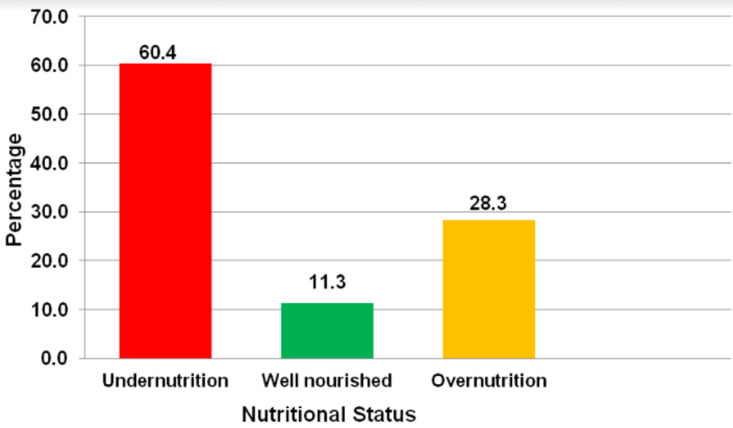
nutritional status among stroke survivors

**Nutritional status of stroke survivors:** majority, 96 (88.7%) of the stroke survivors were malnourished and only 10 (11.3%) were well nourished. Of the survivors who were malnourished, those who were undernourished were 66 (60.4%), while 30 (28.3%) had overnutrition (Figure 1).

The participants who had been discharged over five years were significantly overweight, compared to those who had been discharged for less than 1 year and those discharged between 1-5 years (p = 0.010). There was no significant difference in the mean BMI between survivors from ischaemic stroke and those with haemorrhagic stroke (p = 0.858), as shown in Table 5. There was no significant difference in nutritional status (p = 0.224) between males and females.

**Table 5:** relationship between BMI and demographic characteristics for stroke survivors

Period after discharge	N	Mean BMI (±SD)	p-Value
Less than 1 year	27	22.63±1.9^a,b^	0.010^*^
1-5 years	55	22.08±5.2^b^	
More than 5 years	24	27.29±12.3^c^	
Total	106	23.40±7.2	
**Type of stroke**			
Ischaemic	70	23.49±8.4	0.858
Haemorrhagic	36	23.23±4.4	
Total	106	23.40±7.2	
**Number of stroke episodes**			
Once	97	22.94±15.3	0.091
More than once	9	27.18±18.0	
Total	106		
**Occupation^a^**			
Unemployed	30	22.23±4.5	0.541
Informal	66	23.98±8.3	
Formal	10	23.05±5.9	
Total	106		
**Education level^b^**			
Low	42	23.53±5.9	0.763
Middle	60	23.49±8.2	
High	4	20.77±4.2	
Total	106		
**Gender**			
Male	47	22.43±5.2	0.221
Female	59	24.17±8.5	
Total	106		

^*^Mean values with different superscripts are significantly different at p-value<0.05; occupation^a^: informal=artisans, traders; formal=civil servants and employees of private companies; educational level^b^: low=no school and primary; middle=jhs and shs; high=tertiary education

## Discussion

Two non-modifiable risk factors of stroke are age and sex. In the current study, females formed the higher percentage (56%), while the mean age of all the stroke survivors was 58.5 years. The mean age is consistent with previous research indicating aging as a contributory factor to stroke [14]. The difference to be noted is that while the current study was on stroke survivors undergoing rehabilitation, the previous study was on acute stroke survivors on admission. The finding that participants with middle school education predominated suggests that the low education status could have potentially limited their awareness and knowledge on the disease condition and the nutritional implications [15].

An earlier study carried out at Tema General and Korle Bu Teaching Hospitals in Ghana showed that ischaemic infarction was 78.1%, while haemorrhagic stroke was 18.9% [16]. Though there is high prevalence of ischaemic stroke observed by Donkor *et al*. [16], however, there was a high proportion of the participants with haemorrhagic stroke recorded in this present study. This type of stroke tends to be more serious, so the tendency for its survivors to seek review and rehabilitation would be more compelling and this could contribute to the high prevalence of haemorrhagic stroke among the participants. The period of discharge from the hospital ranged from 6 months to 17 years and the mean period after discharge from the hospital was 3.31 years. This reflects how the disability associated with stroke could persist, to the extent that some of the disabilities could be life-long.

The high level of malnutrition (88.7%) among the participants highlights the need for nutrition intervention. Findings of the study show the double burden of under-nutrition and over-nutrition, with under-nutrition being more prevalent. One factor which could have a major index of malnutrition is the low levels of haemoglobin. Among the five nutritional biomarkers measured, haemoglobin level could provide the best reflection of the poor nutritional state of the stroke survivors. Indeed, 38.7% of the participants had low haemoglobin level which could potentially be indicative of nutritional anaemia. Low level of haemoglobin resulting in anaemia can lead to reduced muscle strength and reduced transportation of oxygen for energy production in mitochondria of body cells, thereby potentially contributing to poor functional recovery among the participants [17]. Studies elsewhere had observed that malnutrition, especially protein-energy malnutrition (PEM), is associated with poor outcome in patients with both ischaemic and haemorrhagic stroke [12,14]. Protein-energy malnutrition alters the expression of plasticity-associated genes that are associated with recovery mechanisms after global ischaemia [15]. This present study has rather shown that though 88.7% of the stroke survivors were malnourished, it was not due to PEM, as a relatively high proportion of the participants had normal levels of albumin and total protein. Albumin is the commonest index used for evaluation of PEM [18].

It is worth noting that of the malnourished participants, the prevalence of undernutrition was 60.4%. This finding falls within the range reported by Bouziana and Tziomalos [19], that undernutrition for stroke survivors living in communities ranges from 6.1% to 62%. A study in Denmark reported that undernutrition was 35% for stroke survivors [12]. In another study in Netherlands, the prevalence of malnutrition among stroke survivors was 73% [17]. The difference in prevalence rates of undernutrition between Denmark and Ghana could be due to the presence of well-organized community stroke rehabilitation services in Denmark, being a developed country, while there are no such community facilities for stroke survivors in Ghana [18]. Additionally, this study has found that close to 3 out of 10 stroke survivors (28.3%) presented with overnutrition, categorized into 16% overweight and 12.3% obese. In another study carried out in Thailand, 41% of stroke survivors were overweight and 18.1% were obese [19]. Over-nutrition as a risk factor for stroke, enhances formation of inflammatory mediators such as c-reactive protein, tumour necrosis factors, intracellular adhesion molecule and interleukin-6 [20]. These inflammatory factors accelerate atherosclerosis, leading to stroke [21]. Other studies have shown that stroke survivors tend to develop obesity after 6 months of the chronic stroke phase, by accumulating excess fat in intramuscular spaces. This is attributed to reduced physical activity due to the physical impact of stroke [22]. The other potential cause could be an improvement in the ability to feed with limited or no capacity for physical activity [23]. Hence, there is need for strategies to address the high rate of overweight/obesity among the participants.

Another indicator of the poor nutritional status was the total lymphocyte count, a haemato-immunological nutrition indicator with 11.3% of the stroke survivors having low level of total lymphocyte count. Low total lymphocyte count tends to indicate a decrease in immunity [24]. This means that these study participants could be prone to risk of infections. Increased risk of infection among these participants could affect functional recovery process and hence, affect their quality of life. The other biomarker that some of the participants showed abnormal high levels was uric acid, for which 25.5% of the participants had levels above the reference range. It has been reported that diets rich in fatty meats and alcohol, increase serum uric acid [25]. Alcohol intake results in elevated uric acid levels due to decreased excretion, as a result of lactate competing for the excretion of urate. Another study has also shown that obesity confers a 3-fold increased risk of hyper-uricaemia. In participants with obesity, insulin resistance stimulates sodium and urate reabsorption in the proximal tubule [26].

It was found that participants who had been discharged for over five years were more overweight than those who had been discharged for a short period of time. This overweight would be attributed to reduced physical activity due to stroke as the fats accumulate in the fatty cells [27]. The long period of time after discharge could have ensured some functional recovery, particularly the ability to feed, leading to some weight gain [28].

## Conclusion

Malnutrition was high (88.7%) among stroke survivors attending a neurology clinic at Komfo Anokye Teaching Hospital. The stroke survivors suffered from both under-nutrition (68.8%) and over-nutrition (31.2%). There was a high prevalence of anaemia among the study participants. The most prevalent biomarker of poor nutrition among the participants is low haemoglobin level. The findings suggest that stakeholders, including clinicians involved in clinical care of stroke survivors in Ghana and other African countries, should include nutrition professionals (clinical nutritionists/dietitians) as part of a multidisciplinary team involved in stroke care to ensure provision of holistic intervention, including nutrition, for optimal outcome. This would help in timely identification and interventions for malnutrition among the stroke survivors to improve recovery and their quality of life.

### What is known about this topic

- Stroke survivors are at risk of malnutrition due to inadequate dietary intake, as a result of neurological disorders causing dysphagia, depression and impaired ability to self-feed and that the burden of stroke is on the increase in low and middle-income countries.

### What this study adds

- This study shows high prevalence of anaemia, under-nutrition and over-nutrition among the stroke survivors which was previously undocumented in the study setting;
- Findings from this study suggest the need for nutrition intervention strategies to address the high burden of malnutrition among the stroke survivors;
- The findings suggest that stakeholders, including clinicians involved in clinical care of stroke survivors in Ghana and other African countries, should include nutritionists and dietitians as part of a multidisciplinary team approach in stroke care to ensure provision of holistic intervention for optimal outcome.

## References

[1] WHO MONICA Project Principal Investigators (1988). The World Health Organization MONICA Project (monitoring trends and determinants in cardiovascular disease): a major international collaboration. Journal of clinical epidemiology.

[2] de Jesús Llibre J, Valhuerdi A, Fernández O, Llibre JC, Porto R, López AM (2010). Prevalence of stroke and associated risk factors in older adults in Havana City and Matanzas Provinces, Cuba (10/66 population-based study). MEDICC Rev.

[3] Sarfo FS, Acheampong JW, Appiah LT, Oparebea E, Akpalu A, Bedu-Addo G (2014). The profile of risk factors and in-patient outcomes of stroke in Kumasi, Ghana. Ghana Med J.

[4] Sarfo FS, Akassi J, Awuah D, Adamu S, Nkyi C, Owolabi M (2015). Trends in stroke admission and mortality rates from 1983 to 2013 in central Ghana. J Neurol Sci.

[5] Sarfo FS, Awuah DO, Nkyi C, Akassi J, Opare-Sem OK, Ovbiagele B (2016). Recent patterns and predictors of neurological mortality among hospitalized patients in Central Ghana. J Neurol Sci.

[6] Finestone HM, Greene-Finestone LS, Wilson ES, Teasell RW (1995). Malnutrition in stroke survivors on the rehabilitation service and at follow-up: prevalence and predictors. Arch Phys Med Rehabil.

[7] Fisher M, Lees K, Spence JD (2006). Nutrition and stroke prevention. Stroke.

[8] Gariballa SE, Parker SG, Taub N, Castleden CM (1998). Influence of nutritional status on clinical outcome after acute stroke. Am J Clin Nutr.

[9] Wasserman S, de Villiers L, Bryer A (2009). Community-based care of stroke survivors in a rural African setting. S Afr Med J.

[10] Creutzfeldt CJ, Holloway RG, Walker M (2012). Symptomatic and palliative care for stroke survivors. J Gen Intern Med.

[11] Agyemang C, Attah-Adjepong G, Owusu-Dabo E, De-Graft Aikins A, Addo J, Edusei AK (2012). Stroke in Ashanti Region of Ghana. Ghana Med J.

[12] Brynningsen PK, Damsgaard EM, Husted SE (2007). Improved nutritional status in elderly patients 6 months after stroke. J Nutr Health Aging.

[13] Teasell RW, Foley NC, Bhogal SK, Speechley MR (2003). An evidence-based review of stroke rehabilitation. Top Stroke Rehabil.

[14] Aquilani R, Sessarego P, Iadarola P, Barbieri A, Boschi F (2011). Nutrition for brain recovery after ischemic stroke: an added value to rehabilitation. Nutr Clin Pract.

[15] Gillum RF, Mussolino ME (2003). Education, poverty and stroke incidence in whites and blacks: the NHANES I Epidemiologic Follow-up Study. J Clin Epidemiol.

[16] Donkor ES, Owolabi MO, Bampoh PO, Amoo PK, Aspelund T, Gudnason V (2014). Profile and health-related quality of life of Ghanaian stroke survivors. Clin Interv Aging.

[17] Chai J, Chu FC, Chow TW, Shum NC (2008). Prevalence of malnutrition and its risk factors in stroke survivors residing in an infirmary. Singapore Med J.

[18] Yoo SH, Kim JS, Kwon SU, Yun SC, Koh JY, Kang DW (2008). Under nutrition as a predictor of poor clinical outcomes in acute ischemic stroke survivors. Arch Neurol.

[19] Bouziana SD, Tziomalos K (2011). Malnutrition in patients with acute stroke. J Nutr Metab.

[20] Prosser-Loose J, Smith ESE, Paterson PG (2011). Experimental model considerations for the study of protein-energy malnutrition co-existing with ischemic brain injury. Curr Neurovasc Res.

[21] Poels BJ, Brinkman-Zijlker HG, Dijkstra PU, Postema K (2006). Malnutrition, eating difficulties and feeding dependence in a stroke rehabilitation centre. Disabil Rehabil.

[22] Oupra R, Griffiths R, Pryor J, Mott S (2010). Effectiveness of supportive educative learning programme on the level of strain experienced by caregivers of stroke survivors in Thailand. Health Soc Care Community.

[23] Vemmos K, Ntaios G, Spengos K, Savvari P, Vemmou A, Pappa T (2011). Association between obesity and mortality after acute first-ever stroke: the obesity-stroke paradox. Stroke.

[24] Haley M, Lawrence CB (2016). Obesity and stroke: can we translate from rodents to patients. J Cereb Blood Flow Metab.

[25] Lansey S, Waslien C, Mulvihill M, Fillit H (1993). The role of anthropometry in the assessment of malnutrition in the hospitalized frail elderly. Gerontology.

[26] Lee JE, Kim YG, Choi YH, Huh W, Kim DJ, Oh HY (2006). Serum uric acid is associated with microalbuminuria in prehypertension. Hypertension.

[27] Roddy E, Choi HK (2014). Epidemiology of gout. Rheum Dis Clin North Am.

[28] Ryan AS, Dobrovolny CL, Smith GV, Silver KH, Macko RF (2002). Hemiparetic muscle atrophy and increased intramuscular fat in stroke survivors. Arch Phys Med Rehabil.

